# Refined genotype–phenotype correlations in neurofibromatosis type 1 patients with NF1 point variants

**DOI:** 10.1136/jmg-2025-110783

**Published:** 2025-08-04

**Authors:** Laurence Pacot, Marinus Blok, Dominique Vidaud, Laura Fertitta, Ingrid Laurendeau, Audrey Coustier, Theodora Maillard, Cécile Barbance, Djihad Hadjadj, Manuela Ye, Dominique Lallemand, Salah Ferkal, Benoit Funalot, Ariane Lunati-Rozie, Bérénice Hebrard, Rakia Bhouri, Liesbeth Spruijt, Didier Bessis, David Geneviève, Vivian Vernimmen, Martinus P G Broen, Sabine Sigaudy, Sylvie Odent, Léna Damaj, Chloé Quélin, Laurent Pasquier, Valérie Layet, Brigitte Gilbert-Dussardier, Gaël Nicolas, Anne-Marie Guerrot, Bruno Leheup, Anne-Claire Bursztejn, Florence Petit, Odile Boute-Bénéjean, Yline Capri, Anne Guimier, Stanislas Lyonnet, Genevieve Baujat, Emmanuelle Bourrat, Bertrand Isidor, Mathilde Nizon, Sébastien Barbarot, Annick Toutain, Sophie Blesson, Julien Van-Gils, Fanny Morice-Picard, Séverine Audebert-Bellanger, Juliette Mazereeuw-Hautier, Alban Ziegler, Yves Alembik, Juliette Piard, Elise Brischoux-Boucher, Léa Guerrini-Rousseau, Julia Morera, Véronique Paquis-Flucklinger, Bruno Delobel, Jean-Luc Alessandri, Béatrice Parfait, on behalf of NF-France network, Henri Adamski, Pierre Wolkenstein, Eric Pasmant

**Affiliations:** 1 Institut Cochin, Inserm U1016, CNRS UMR8104, Université Paris Cité, CARPEM, Paris, France; 2 Fédération de Génétique et Médecine Génomique, DMU BioPhyGen, Hôpital Cochin, AP-HP.Centre-Université Paris Cité, Paris, France; 3 Department of Clinical Genetics, Maastricht University Medical Center, Maastricht, The Netherlands; 4 GROW-School for Oncology and Reproduction, Maastricht University, Maastricht, The Netherlands; 5 Department of Dermatology, Hôpital Henri Mondor, Assistance Publique-Hôpital Paris (AP-HP), Créteil, France; 6 INSERM U955, Université Paris Est Créteil (UPEC), Créteil, France; 7 Department of Genetics, AP-HP (Assistance Publique-Hôpitaux de Paris), Henri Mondor University Hospital, Créteil, France; 8 Department of Ophthalmology, Centre Hospitalier Intercommunal de Créteil (CHIC), Créteil, France; 9 Department of Clinical Genetics, Radboud University Medical Centre, Nijmegen, The Netherlands; 10 Department of Dermatology and Reference Center for Rare Skin Diseases MAGEC-Sud Montpellier, Filière Maladies Rares Dermatologiques (FIMARAD), Saint-Eloi Hospital, and University of Montpellier, Montpellier, France; 11 Inserm U1183, Department of Clinical Genetics, Reference center for rare disease developmental anomaly and malformative syndrome, CHU Montpellier, and Montpellier University, Montpellier, France; 12 Department of Neurology, GROW School for Oncology and Reproduction, Maastricht University Medical Centre, Maastricht, The Netherlands; 13 Department of Medical Genetics, Children's Hospital La Timone, Assistance Publique des Hôpitaux de Marseille, Marseille, France; 14 Service de génétique clinique, CLAD Ouest, CHU Rennes, Hôpital Sud, Rennes, France; 15 Consultations de Génétique, Groupe Hospitalier du Havre, Le Havre, France; 16 Service de Génétique, CHU de Poitiers, Poitiers, France; 17 Department of Genetics and reference center for developmental abnormalities, Univ Rouen Normandie, Normandie Univ, Inserm U1245 and CHU Rouen, Rouen, France; 18 Service de Génétique Médicale, Hôpitaux de Brabois, CHRU de Nancy, Vandoeuvre-lès-Nancy, France; 19 Department of Dermatology, CHRU Nancy, Vandoeuvre-lès-Nancy, France; 20 Clinique de Génétique, Centre de Référence Anomalies du Développement, Univ. Lille, CHU Lille, Lille, France; 21 UF de Génétique Clinique, CHU Robert Debré, Paris, France; 22 Service de Médecine Génomique des Maladies Rares et Institut Imagine UMR-1163 Inserm, Université Paris Cité, Hôpital Necker-Enfants Malades, Assistance Publique des Hôpitaux de Paris, Paris, France; 23 Department of Dermatology, MAGEC-Nord Hôpital Saint Louis, Assistance Publique des Hôpitaux de Paris (AP-HP), Paris, France; 24 Medical Genetics Department, CHU de Nantes, Hôtel Dieu Hospital, Nantes, France; 25 Department of Dermatology, CHU Nantes, INRAE, UMR 1280, PhAN, Nantes University, Nantes, France; 26 Department of Genetics, Bretonneau University Hospital, Tours, France; 27 UMR 1253, iBrain, University of Tours, Inserm, Tours, France; 28 Département de Génétique Médicale, Centre Hospitalier Universitaire de Bordeaux, Bordeaux, France; 29 Pediatric Dermatology Unit, National Center for Rare Skin Disorders, University Hospital of Bordeaux, Bordeaux, France; 30 Service de Pédiatrie et de Génétique Médicale, CHRU Morvan, Brest, France; 31 Service de Dermatologie, Centre de Référence des Maladies rares de la peau, Hôpital Larrey, Toulouse, France; 32 Department of Genetics, University Hospital of Toulouse, Toulouse, France; 33 Service de Génétique Médicale, Institut de Génétique Médicale d'Alsace, Hôpitaux Universitaires de Strasbourg, Strasbourg, France; 34 Centre de Génétique Humaine, Centre Hospitalier Universitaire de Besançon, Besançon, France; 35 UMR1231 GAD, Inserm, Université de Bourgogne, Dijon, France; 36 Department of Children and Adolescents Oncology, Gustave Roussy, Université Paris-Saclay, Villejuif, France; 37 Department of Endocrinology and Diabetology, CHU Côte de Nacre, Caen, France; 38 Inserm U1081, CNRS UMR7284, IRCAN, Université Côte d'Azur, CHU de Nice, Nice, France; 39 Service de génétique médicale, GH de l'Institut Catholique de Lille, Lille, France; 40 service de pédiatrie, CHU Féleix Guyon, Saint-Denis, France; 41 Genetics Department, Institut Curie, Paris, France

**Keywords:** Genetic Diseases, Inborn

## Abstract

**Background:**

Neurofibromatosis type 1 (NF1) is one of the most frequent genetic disorders. NF1 is caused by dominant loss-of-function pathogenic variants (PVs) of the tumour-suppressor gene *NF1*, which encodes neurofibromin, a negative regulator of rat sarcoma proteins. NF1 is an autosomal dominant disorder with complete penetrance, but a highly variable expression. Identification of genotype–phenotype correlations is challenging because of the wide clinical variability, the progressive nature of the disorder and the extreme diversity of the mutation spectrum. Only a few *NF1* point variants have been associated with a specific phenotype in NF1 patients.

**Methods:**

We investigated a large, well-phenotyped NF1 cohort.

**Results:**

We report analyses of genotype-phenotype correlations in 112 NF1 patients with specific *NF1* point variants: p.Arg1809 missense variants were associated with a mild form of NF1 (n=24), while a more severe phenotype was associated with codons 844–848 (n=27), p.Arg1276 (n=25) and p.Lys1423 (n=35) missense variants. We describe a new correlation for p.Arg1204 missense variants (n=11), with no neurofibroma observed in patients. Functional studies will be critical for drawing conclusions on the potential hypomorphic or dominant-negative effects of these variants.

**Conclusion:**

The current data confirms several genotype-phenotype correlations in NF1, which may be relevant to the management and surveillance of NF1 patients with specific *NF1* PVs.

**WHAT IS ALREADY KNOWN ON THIS TOPIC**

- Neurofibromatosis type 1 (NF1) is a rare syndrome disorder caused by *NF1* pathogenic variants, leading to pleiotropic neurocutaneous symptoms with considerable clinical variability.
- Prior studies have established some genotype–phenotype relationships, but these findings are based on small cohorts, and certain correlations remain to be confirmed or discovered.

**WHAT THIS STUDY ADDS**

- This study confirms that p.Met1149 and p.Arg1809 missense variants are associated with a mild NF1 phenotype, whereas more severe manifestations correlate with p.Arg1276, p.Lys1423 and the 844–848 codon missense variants.
- A correlation involving p.Arg1204 variants, with no neurofibromas, was observed in affected individuals.

**HOW THIS STUDY MIGHT AFFECT RESEARCH, PRACTICE OR POLICY**

- Well-defined functional modelling studies will be critical for genotype–phenotype correlations confirmation in NF1.
- Confirmed genotype–phenotype relationships may lay a foundation for personalised genotype-guided care strategies for NF1 patients.

## Introduction

 Neurofibromatosis type 1 (NF1; MIM 162200) is one of the most prevalent autosomal dominant disorders. NF1 is caused by dominant loss-of-function pathogenic variants (PVs) of the tumour-suppressor gene *NF1*, which encodes neurofibromin, a critical negative regulator of rat sarcoma (RAS)-mitogen-activated protein kinase (MAPK) signalling pathways.^1^ NF1 is a tumour predisposition syndrome and the most common NF1-associated tumours are benign peripheral nerve tumours that may be cutaneous neurofibromas (cNFs), subcutaneous neurofibromas (scNFs) or plexiform neurofibromas (pNFs). Patients with NF1 are at increased risk of developing a variety of tumours, including malignant peripheral nerve sheath tumours (MPNSTs), optic pathway gliomas (OPGs), astrocytic neoplasms, juvenile myelomonocytic leukaemias, gastrointestinal stromal tumours, breast cancers, pheochromocytomas, duodenal carcinoids, glomus tumours, juvenile xanthogranulomas and rhabdomyosarcomas. NF1 is a multisystemic condition characterised by café-au-lait spots (CALS), axillary and inguinal skinfold freckling, Lisch nodules of the iris and skeletal abnormalities (eg, pseudarthrosis).^2^ Typically, identification of affected individuals has relied on clinical assessment and diagnosis through standardised NIH (*National Institutes of Health*) criteria.^3^

NF1 is a progressive disorder with features that typically develop with age. Although NF1 is a simply determined Mendelian disorder with complete penetrance, it is characterised by highly interfamilial and intrafamilial variable expression.^4^ For many genetic conditions, the correlation of a specific DNA change to disease features is complex. Multiple factors may affect phenotype, including age-dependent manifestations, allelic and non-allelic heterogeneity, the timing and nature of the second hit in specific cells, wild-type allele, modifier genes, environment and stochastic factors. A specific phenotype is determined by the interaction of these factors. NF1 clinical outcome cannot be predicted by the type of *NF1* PVs and evidence for the existence of modifier genes has been obtained in large familial studies.^5^,^8^

Of the 4481 different constitutional *NF1* PVs identified (*The Human Gene Mutation Database Professional 2024.2*), only a few point *NF1* PVs^9^,^13^ and complete deletions of the *NF1* locus^14^,^17^ have been associated with a specific phenotype in NF1 patients.^18^ Each of these accounts for a small proportion of affected patients, totalling ~10-15% of people with NF1.

c.2970_2972del p.Met992del and p.Arg1809 missense variants were associated with a milder phenotype,^9 10^ whereas missense variants in *NF1* codons 844–848 were described to confer greater risks of severity.^12^ The other reported variants associated with genotype-phenotype correlation include c.3112A>G p.Arg1038Gly,^19^ missense or splice-site variants in familial spinal neurofibromatosis, PVs in 5′ tertile in patients with OPGs,^20^,^22^ non-truncating PVs in patients with pulmonary stenosis,^23^ missense PVs in the cysteine/serine-rich domain,^24^ missense PVs affecting p.Met1149, p.Arg1276 and p.Lys1423,^11^ and variants responsible for in-frame skipping of exon 24.^25^ In a study including 493 patients, 62 of whom had a congenital heart defect (with 23 pulmonic stenosis), Pinna *et al*^26^ demonstrated an association between the presence of pathogenic non-truncating (missense and inframe) *NF1* variants and heart defects. Interestingly, some variants were previously associated with more frequent pulmonic stenosis (Met992del, Met1149Val and Lys1423Glu). Some of the variants were localised in the GTPase (guanosine triphosphate phosphatase)-activating protein-related domain (GRD) region of neurofibromin (Leu1196Arg, Ala1387Asp, Arg1391Ser, Lys1436Glu, Glu1438del and Gly1498Glu). The pulmonic stenosis was associated with Noonan-like features in 19/20 patients. In a 2022 publication, Ho *et al*^27^ compared the clinical and molecular data of 746 patients aged over 8 years. They observed that neurofibromas were less frequent in patients with non-truncating variants, while macrocephaly, cardiac anomalies or pulmonary valve stenosis were more frequently observed. Furthermore, carriers of non-truncating variants located in the GRD had significantly fewer cNFs compared with carriers of non-truncating variants located outside the GRD.

**Figure 1:**
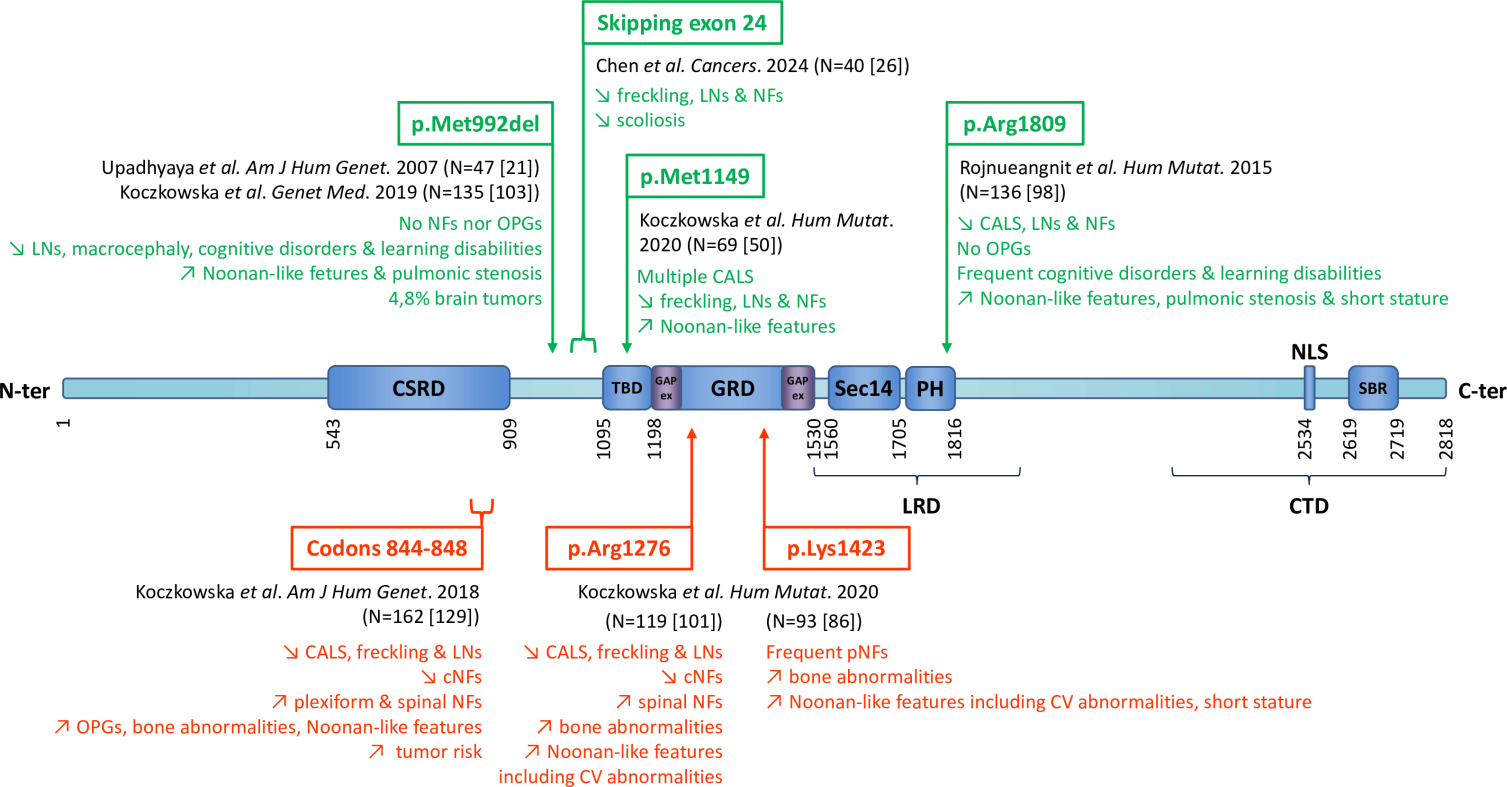
Location of *NF1* gene point variants associated with a specific form of the disease. N indicates the number of patients described in each study, with the number of families involved in square brackets. Arrows indicate symptoms significantly more frequently (↗) or more rarely (↘) observed in the different groups, compared with the overall population of NF1 patients, at the 5% threshold. Numbers indicate the position of neurofibromin amino acids. CALS, café-au-lait spots; cNFs, cutaneous neurofibromas; CSRD, cysteine/serine-rich domain; CTD, C-terminal domain; CV, cardiovascular; GRD, rat sarcoma-GAP (GTPase-activating protein)-related domain; LNs: Lisch nodules; LRD, leucine-rich domain; NFs, neurofibromas; NLS, nuclear localisation signal; OPGs, optic pathway gliomas; pNFs, plexiform neurofibromas; SBR, syndecan-binding region; Sec14-PH, *Saccharomyces cerevisiae* phosphatidylinositol transfer protein-like domain–pleckstrin homology-like domain; TBD, tubulin-binding domain.

Several other studies have failed to establish a significant genotype-phenotype correlation, despite large cohorts.^28^,^31^ Figure 1 and online supplemental table 1 summarise the main genotype-phenotype correlations established for point variants in *NF1*. In the light of these extensive and sometimes contradictory studies of genotype–phenotype correlations for *NF1* point variants, we investigated a large, well-phenotyped NF1 cohort. Here, we present analyses of genotype–phenotype correlations associated with *NF1* point variants.

## Results

### Cohort

**Figure 2:**
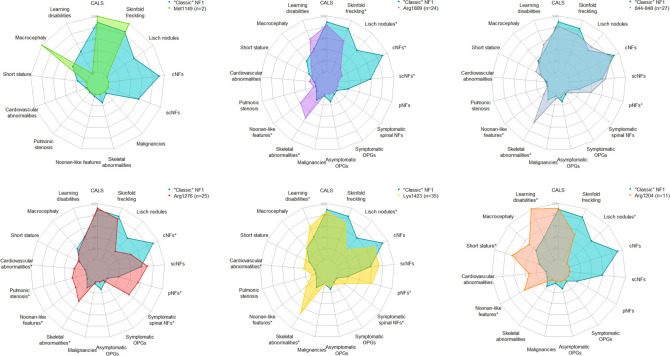
Radar plot of the frequency of the main clinical features in the present cohort and the ‘classic’ NF1 cohort. Frequency of features with a white circle (°) or an asterisk (*) significantly differs between the two cohorts, respectively, only before or after correction for multiple testing with the Benjamini-Hochberg procedure. CALS, café-au-lait spots; cNFs, cutaneous neurofibromas; NF1, neurofibromatosis type 1; OPGs, optic pathway gliomas; pNFs, plexiform neurofibromas; scNFs, subcutaneous neurofibromas.

A total of 124 patients with specific heterozygous *NF1* (NM_000267.3) point variants were included in the study. Patient clinical data are summarised in tables1^4^. Online supplemental table 1 shows the comparison of the frequency of the main clinical signs between patients carrying the different variants and the ‘classic’ NF1 population, summarised in the radar diagrams in figure 2.

**Table 1:** Clinical data in NF1 patients with Arg1809 missense variants

	Age: 0–8	Age: 9–18	Age>18	Total n/N*	Total %
Age range (years)	1–8	10–18	24–57	1–57	
Median age (years)	5	13	39	16	
Number of individuals (index cases:relatives)	5:3	5:1	3:7	13:11	
Male:female	7:1	4:2	6:4	17:7	
Clinical diagnostic criteria fulfilled considering family history	6/8	6/6	8/10	20/24	83%
Clinical diagnostic criteria fulfilled regardless of family history	5/8	6/6	7/10	18/24	75%
Café-au-lait spots†	6/8	6/6	10/10	22/24	92%
1-5			2/10		
6-100	5/8	6/6	5/10		
>100	1/8		2/10		
Not quantified			1/10		
Freckling	4/8	4/5	6/10	14/23	61%
Blue-red macules	0/8	0/6	0/8	0/22	0%
Lisch nodules	0/7	1/5	2/7	3/19	16%
Unilateral			1/7		
Bilateral					
Not specified		1/5	1/7		
Cutaneous neurofibroma‡	0/8	0/6	1/10	1/24	4%
Subcutaneous neurofibroma‡	0/8	0/6	0/10	0/24	0%
Deep neurofibroma	0/7	0/5	0/8	0/20	0%
Plexiform neurofibroma	0/2	ND	0/2	0/4	0%
Spinal neurofibroma	0/2	ND	0/2	0/4	0%
Optic pathway glioma§	0/6	0/4	0/3	0/13	0%
Other tumours	0/7	0/6	0/9	0/22	0%
Musculoskeletal abnormalities¶	2/7	5/6	3/9	10/22	45%
Scoliosis	0/7	2/6	3/9		
*Pectus* abnormalities	3/7	4/6	0/9		
Noonan-like features‡‡	4/8	3/6	2/9	9/23	39%
Short stature (<2 SD)	2/8	0/6	0/6	2/20	10%
Macrocephaly (>2 SD)	1/7	0/5	3/6	4/18	22%
Neurological abnormalities**	3/7	4/6	1/8	8/21	38%
UBOs	0/7	1/6	1/8		
Cognitive disorders and/or learning difficulties	6/8	5/6	3/8	14/22	64%
Cardiovascular abnormalities††	1/7	0/4	1/8	2/19	11%
Pulmonic stenosis	1/7		0/8		

* n=number of patients fulfilling the criterion; N=total number of patients for whom data were available.

† All sizes.

‡ Including non-histologically confirmed neurofibromas.

§ Optic pathways gliomas identified by brain MRI or CT scan.

¶ Musculoskeletal abnormalities including scoliosis, *pectus excavatum*, *pectus carinatum*, short left leg, scaphocephaly.

** Neurological abnormalities including global hypotonia, headache, UBOs, enlarged lateral ventricles.

†† Cardiovascular abnormalities including pulmonary valve stenosis, multiple ventricular septal defects, mitral and pulmonic insufficience.

‡‡ Noonan-like features including low implanted ears, hypertelorism, *pterygium colli*, median facial hypoplasia, ptosis, proptosis.

ND, not documented; NF1, neurofibromatosis type 1; UBOs, unidentified bright objects (hyperintense regions seen on T2-weighted magnetic resonance brain scans).

**Table 2:** Clinical data in NF1 patients with codon 844-848 missense variants

	Age: 0–8	Age: 9–18	Age>18	Total n/N*	Total %
Age range (years)	1–7	10–17	25–65	1–65	
Median age (years)	3	14	39	26	
Number of individuals (index cases:relatives)	4:3	2:3	11:4	17:10	
Male:Female	1:6	5:0	11:4	17:10	
Clinical diagnostic criteria fulfilled considering family history	6/7	5/5	15/15	26/27	96%
Clinical diagnostic criteria fulfilled regardless family history	4/7	5/5	14/15	23/27	85%
Café-au-lait spots†	7/7	5/5	15/15	27/27	100%
1–5		1/5	4/15		
6–100	7/7	4/5	9/15		
>100					
Not quantified			2/15		
Freckling	3/7	4/5	12/14	19/26	73%
Blue-red macules	0/7	0/3	1/11	1/21	5%
Lisch nodules	2/5	3/3	5/7	10/15	67%
Unilateral		1/3			
Bilateral	1/5		2/7		
Not specified	1/5	2/3	3/7		
Cutaneous neurofibroma‡	0/7	0/4	12/14	12/25	48%
1					
2–9			1/14		
10–99			7/14		
>100			1/14		
Not quantified			3/14		
Subcutaneous neurofibroma‡	0/7	2/5	9/14	11/26	42%
1			2/14		
2–9		1/5	3/14		
10–99		1/5	1/14		
>100			1/14		
Not quantified			2/14		
Deep neurofibroma	0/6	ND	3/7	3/13	23%
Plexiform neurofibroma	0/3	0/2	6/13	6/18	33%
Spinal neurofibroma	0/2	0/2	1/9	1/13	8%
Symptomatic			1/9		
Asymptomatic					
Not specified					
Optic pathway glioma§	0/6	0/2	1/9	1/17	6%
Other tumours¶	1/7	0/5	3/13	4/25	16%
MPNSTs			1/13		
Musculoskeletal abnormalities**	1/7	3/5	9/14	13/26	50%
Scoliosis	0/7	3/5	8/14		
*Pectus* abnormalities	0/7	0/5	0/14		
Noonan-like features	0/7	2/5	1/12	3/24	13%
Short stature (<2 SD)	0/7	3/5	3/12	6/24	25%
Macrocephaly (>2 SD)	1/6	1/5	4/9	6/20	30%
Neurological abnormalities††	4/7	2/5	9/14	15/26	58%
UBOs	2/7	1/5	3/14		
Cognitive disorders and/or learning difficulties	4/7	3/5	7/14	14/26	52%
Cardiovascular abnormalities‡‡	0/6	0/4	1/12	1/22	5%
Pulmonic stenosis			0/3		

* n=number of patients fulfilling the criterion; n=total number of patients for whom data were available.

† All sizes.

‡ Including non-histologically confirmed neurofibromas.

§ Optic pathways gliomas identified by brain MRI or CT-scan.

¶ Other tumours including MPNST, juvenile xanthogranuloma, pheochromocytoma, left temporal pilocytic astrocytoma, eyelid xanthogranuloma, bilateral mammary carcinoma.

** Musculoskeletal abnormalities including scoliosis, pseudarthrosis, *genu recurvatum*, *valgus* feet.

†† Neurological abnormalities including headaches, UBOs, epilepsy, spinal cord compression.

‡‡ Cardiovascular abnormalities including hypertension.

MPNSTs, malignant peripheral nerve sheath tumours; ND, not documented; NF1, neurofibromatosis type 1; UBOs, unidentified bright objects (hyperintense regions seen on T2-weighted magnetic resonance brain scans).

**Table 3:** Clinical data in NF1 patients with Arg1276 missense variants

	Age: 0–8	Age: 9–18	Age>18	Total n/N*	Total %
Age range (years)	1–8	12–17	19–60	1–60	
Median age (years)	5	17	29	23	
Number of individuals (index cases:relatives)	5:1	4:1	13:1	22:3	
Male:female	4:2	3:2	6:8	13:12	
Clinical diagnostic criteria fulfilled considering family history	5/6	5/5	14/14	24/25	96%
Clinical diagnostic criteria fulfilled regardless of family history	5/6	4/5	14/14	23/25	92%
Café-au-lait spots†	6/6	5/5	14/14	25/25	100%
1–5	1/6		1/14		
6–100	4/6	4/5	13/14		
>100					
Not quantified	1/6	1/5			
Freckling	4/5	4/5	11/14	19/24	79%
Blue-red macules	0/5	0/4	1/12	1/21	5%
Lisch nodules	0/4	1/3	2/4	3/11	27%
Unilateral					
Bilateral		1/3			
Not specified			2/4		
Cutaneous neurofibroma‡	1/6	0/4	6/13	7/23	30%
1			1/13		
2–9	1/6		2/13		
10–99			2/13		
>100			1/13		
Not quantified					
Subcutaneous neurofibroma‡	0/5	1/5	9/13	10/23	43%
1		1/5			
2–9			3/13		
10–99			5/13		
>100					
Not quantified			1/13		
Deep neurofibroma	1/3	0/2	7/10	8/15	53%
Plexiform neurofibroma	1/2	0/1	6/10	7/13	54%
Spinal neurofibroma	ND	ND	3/6	3/6	50%
Symptomatic			3/6		
Asymptomatic					
Not specified					
Optic pathway glioma§	0/3	0/4	0/9	0/16	0%
Other tumours¶	0/4	0/4	1/14	1/22	5%
MPNSTs			0/14		
Musculoskeletal abnormalities**	3/6	3/4	5/14	11/24	46%
Scoliosis	0/6	0/4	3/14		
*Pectus* abnormalities	2/6	3/4	1/14		
Noonan-like features	4/5	1/5	2/14	7/24	29%
Short stature (<2 SD)	1/5	2/5	1/12	4/22	18%
Macrocephaly (>2 SD)	1/4	0/3	4/10	5/17	29%
Neurological abnormalities††	1/5	2/4	6/14	9/23	39%
UBOs	1/5	0/4	3/14		
Cognitive disorders and/or learning difficulties	3/6	4/5	4/14	11/25	44%
Cardiovascular abnormalities‡‡	2/5	2/5	2/13	6/23	26%
Pulmonic stenosis	2/5	1/5	1/5		

* n=number of patients fulfilling the criterion; N=total number of patients for whom data were available.

† All sizes.

‡ Including non-histologically confirmed neurofibromas.

§ Optic pathways gliomas identified by brain MRI or CT scan.

¶ Other tumours including central giant cell granuloma.

** Musculoskeletal abnormalities including scoliosis, kyphosis, *pectus excavatum*, *pectus carinatum*, *genu varum*, vertebral dysplasia, long bone dysplasia, agenesis of distal end of ulna, radius curvature.

†† Neurological abnormalities including headaches, UBOs, Chiari malformation type 1, spinal cord compression, partial mesial temporal seizures.

‡‡ Cardiovascular abnormalities including pulmonary valve stenosis, atrial septal defect, hypertrophic cardiomyopathy, hypertension.

MPNSTs, malignant peripheral nerve sheath tumours; ND, not documented; NF1, neurofibromatosis type 1; UBOs, unidentified bright objects (hyperintense regions seen on T2-weighted magnetic resonance brain scans).

**Table 4:** Clinical data in NF1 patients with Lys1423 missense variants

	Age: 0–8	Age: 9–18	Age>18	Total n/N*	Total %
Age range (years)	1–7	9–18	19–68	1–68	
Median age (years)	3	13	34	18	
Number of individuals (index cases:relatives)	9:0	8:2	12:4	29:6	
Male:female	4:5	5:5	4:12	13:22	
Clinical diagnostic criteria fulfilled considering family history	8/9	9/10	16/16	33/35	94%
Clinical diagnostic criteria fulfilled regardless of family history	7/9	9/10	16/16	32/35	91%
Café-au-lait spots†	9/9	10/10	15/16	34/35	97%
1–5			4/16		
6–100	9/9	9/10	11/16		
>100					
Not quantified		1/10			
Freckling	3/9	8/10	13/15	24/34	71%
Blue-red macules	0/8	2/9	4/15	6/32	19%
Lisch nodules	0/6	1/10	7/11	8/27	30%
Unilateral		1/10	2/11		
Bilateral					
Not specified			5/11		
Cutaneous neurofibroma‡	0/9	4/9	12/16	16/34	47%
1		1/9			
2–9		2/9	5/16		
10–99		1/9	2/16		
>100			4/16		
Not quantified			1/16		
Subcutaneous neurofibroma‡	0/9	4/9	11/15	15/33	45%
1					
2–9		3/9	4/15		
10–99		1/9	5/15		
>100					
Not quantified			2/15		
Deep neurofibroma	0/7	1/6	3/10	4/23	17%
Plexiform neurofibroma	0/4	7/8	5/11	12/23	52%
Spinal neurofibroma	0/3	1/4	1/7	2/14	14%
Symptomatic		1/4	1/7		
Asymptomatic					
Not specified					
Optic pathway glioma§	3/9	1/8	0/6	4/23	17%
Symptomatic OPGs	2/9				
Asymptomatic OPGs					
Other tumours¶	0/6	1/8	2/14	3/28	11%
MPNSTs		0/8	0/14		
Musculoskeletal abnormalities**	1/8	4/9	12/14	17/31	55%
Scoliosis	0/8	3/9	11/14		
*Pectus* abnormalities	0/8	1/9	1/14		
Noonan-like features	3/5	2/7	2/15	7/27	26%
Short stature (<2 SD)	1/8	1/9	5/14	7/31	23%
Macrocephaly (>2 SD)	1/7	5/9	3/11	9/27	33%
Neurological abnormalities††	5/9	5/10	9/15	19/34	56%
UBOs	4/9	2/10	3/15		
Cognitive disorders and/or learning difficulties	5/8	7/10	9/15	21/33	64%
Cardiovascular abnormalities‡‡	3/6	1/7	4/14	8/27	30%
Pulmonic stenosis	1/4	0/5	0/6		

* n=number of patients fulfilling the criterion; N=total number of patients for whom data were available.

† All sizes.

‡ Including non-histologically confirmed neurofibromas.

§ Optic pathways gliomas identified by brain MRI or CT scan.

¶ Other tumours including pheochromocytoma, xanthogranuloma, ductal carcinoma.

** Musculoskeletal abnormalities including scoliosis, kyphoscoliosis, hyperlordosis, *pectus excavatum*, *pectus carinatum*, brachydactyly, sphenoid wing dysplasia, plagiocephaly, left anterior costal dystrophy.

†† Neurological abnormalities including headaches, UBOs, epilepsy, hypotonia, hearing loss, hydrocephalus, Chiari malformation, left internal carotid hypoplasia.

‡‡ Cardiovascular abnormalities including pulmonary valve stenosis, aortic valve stenosis, hypertension, dilated heart disease, aortic regurgitation, patent foramen ovale, ventricular septal defect.

MPNSTs, malignant peripheral nerve sheath tumours; NF1, neurofibromatosis type 1; OPGs, optic pathway gliomas; UBOs, unidentified bright objects (hyperintense regions seen on T2-weighted magnetic resonance brain scans).

### Missense variants at p.Met1149

We observed two index cases with the variant c.3447G>A p.Met1149Ile. The two patients had numerous CALS, but no neurofibromas were observed. However, since only two patients were identified in our cohort, no statistical comparison was made with the reference cohort (online supplemental table 2 and figure 2).

### Missense variants at p.Arg1809

The following variants were represented in the cohort: c.5425C>T p.Arg1809Cys, 11 index cases and 10 relatives; c.5425C>G p.(Arg1809Gly), 1 index case and his mother; c.5425C>A p.(Arg1809Ser), 1 index case. These patients with p.Arg1809 missense variants had significantly fewer freckling (p=0.020), Lisch nodules (p<0.001), cNFs (p=1.5×10^−7^) and scNFs (p=0.00096), and more frequent bone abnormalities (p=0.0028) and Noonan-like features (p=5.4.10^−7^), when compared with the ‘classic NF1’ cohort (table 1 and figure 2).

### Missense variants at codons 844–848

These variants implicated various codons: c.2531T>C p.Leu844Pro, two index cases and one relative; c.2533T>C p.Cys845Arg, one index case; c.2540T>C p.Leu847Pro, eight index cases and two relatives; c.2543G>A p.Gly848Glu, three index cases and seven relatives; c.2542G>C p.Gly848Arg, three index cases. Reported neurological abnormalities mainly consisted of headaches (7/26) or UBOs (6/26). Missense variants of amino acids 844–848 were associated with a significant over-representation of bone anomalies (p=6.9×10^−5^). A significant difference before correction for multiple testing was observed for pNFs (p=0.047 and p=0.22, respectively, before and after correction), scoliosis (p=0.0057 and p=0.051, respectively, before and after correction) and Noonan-like features (p=0.049 and p=0.22, respectively, before and after correction). Short stature was frequent (6/24, 25%), although the difference was not significant compared with the reference cohort (p=0.26 without correction) (table 2 and figure 2).

### Missense variants at p.Arg1276

For this position in neurofibromin, we detected three distinct variants: c.3827G>A p.Arg1276Gln, 19 index cases and two relatives; c.3826C>G p.Arg1276Gly, two index cases and one relative; c.3827G>T p.Arg1276Leu, one index case. The p.Arg1276 missense variants were associated with a more severe phenotype, with significantly more frequent pNFs (p=0.022), symptomatic spinal NFs (p=0.00054), bone abnormalities (p=0.010), Noonan-like features (p=0.00021), cardiovascular abnormalities (p=0.00072) and pulmonic stenosis (p=0.00021). This group also had significantly fewer cNFs (p=0.00050) (table 3 and figure 2).

### Missense variants at p.Lys1423

The following variants were represented in the cohort: c.4267A>G p.Lys1423Glu variant, 27 index cases and 6 relatives; c.4268A>T p. Lys1423Met, 1 index case; c.4269G>C p.(Lys1423Asn), 1 index case. In this group of 35 patients, we observed a significantly higher frequency of pNFs (p=0.00015), symptomatic spinal NFs (p=0.042), bone anomalies (p=2.3×10^−7^), including scoliosis (p=0.00015), Noonan-like features (p=0.00015) and cardiovascular anomalies (p=0.00015). There were also significantly fewer Lisch nodules (p=0.008). Although not significant, we observed cognitive disorders or learning difficulties more often (p=0.046 and p=0.10, respectively, before and after correction) and there was a tendency for less cNFs (p=0.059 and p=0.11, respectively, before and after correction) (table 4 and figure 2).

### Missense variants at p.Arg1204

We identified eleven patients with missense variants at this position in neurofibromin: nine had the c.3610C>G p.(Arg1204Gly) variant (seven index cases and two relatives) and two had the c.3610C>T p.Arg1204Trp variant (one index case and his grandfather). These patients had no neurofibroma, but statistical analyses were not significant as only patients over 18 years old were considered for these symptoms and exhaustive data were not always available for all patients. They presented more frequently with learning disabilities, Noonan-like features and short stature (p=0.046 after correction). They developed Lisch nodules less frequently (p=0.029 and 0.12, respectively, before and after correction) (online supplemental table 3 and figure 2).

## Discussion

Phenotypic variability in NF1 is a major challenge for patient care and genetic counselling. Recent large-scale international studies have expanded our understanding of genotype–phenotype correlations in NF1.

Our genotype–phenotype correlation analysis, conducted in a large NF1 cohort, largely corroborates findings from prior international studies. Specifically, p.Met1149 and p.Arg1809 missense variants are associated with a mild NF1 phenotype, whereas more severe manifestations correlate with p.Arg1276, p.Lys1423 and the 844–848 codon missense variants. These variants respectively represented 0.09% (3/3479), 0.52% (18/3479), 0.69% (24/3479), 0.98% (34/3479) and 0.55% (19/3479) of all positive index cases in our cohort. We also propose a new correlation involving p.Arg1204 variants, with no neurofibromas observed in affected individuals.

The p.Met992del variant has long been associated with a mild phenotype including CALS and absence of neurofibromas.^10 32^ We did not detail it here as it was already published,^32^ but our data aligned with prior studies suggesting hypomorphic effects. The high frequency of p.Met992del in databases may be due to a mutational hotspot and reduced negative selection, given the mild phenotype.

Five different p.Arg1809 missense variants have been described, most frequently p.Arg1809Cys.^13 33 34^ Previous studies, including large cohorts, consistently showed these variants were associated with few or no neurofibromas, no optic gliomas and frequent Noonan-like features. Our cohort confirmed this pattern, including a potential association with learning disabilities (see table 1; 64%). None of the carrier patients developed neurofibromas, OPGs or other tumours (table 1 and figure 2). However, we observed significantly more dysmorphic Noonan-like features, as well as bone abnormalities. There was no excess of short stature, nor pulmonary valve stenosis. Functional data suggested that these variants act as hypomorphs, with partial impact on RAS-MAPK signalling.^33 35^

In 2018, Koczkowska *et al* showed an association between missense variants affecting neurofibromin codons 844–848 and a specific clinical presentation with rare cNFs and freckling, and an increased occurrence of pNFs and spinal neurofibromas, skeletal abnormalities, OPGs and cancers.^12^ The clinical features of some patients suggested an alternative form of NF1, called familial spinal neurofibromatosis (MIM #162210)^36^ due to the presence of numerous spinal neurofibromas, with or without other features suggestive of NF1; 12 out of 14 patients carrying missense variants of p.Gly848 had spinal neurofibromas, and a much lower proportion of skin manifestations such as TCLs, frecklings or cNFs was observed for this codon compared with other missense variants in this 844–848 region. The variants’ heterogeneity raises the question of the involvement of each of these variants individually in the overall phenotype. In our cohort, the phenotype associated with missense variants in codons 844–848 showed some similarities with data from the literature (table 2 and figure 2).^12^ Plexiform neurofibromas were more frequent (6/15, 40%) compared with the reference cohort (120/648, 18%), as previously reported (36/92, 39%). Similarly, Noonan-like features and bone abnormalities, including scoliosis, were more frequently described in these patients. However, we did not observe a rarer occurrence of cNFs in patients carrying these variants, or even of an increased risk of OPGs or malignant tumours. These discrepancies could be due to the heterogeneity of the variants affecting codons 844–848. Another study suggested an increased breast cancer risk associated with missense variants affecting amino acid Leu847.^24^ In the present study, one patient had developed bilateral mammary carcinoma, diagnosed at the age of 65. An in vitro study suggested a significant functional variability in the 844–848 region: for instance, the p.Leu847Pro variant was found to be associated with a high activation of the RAS activity, while the p.Gly848Arg variant had no discernible in vitro effect.^37^ Interestingly, a mouse model previously suggested the absence of OPGs associated with the p.Gly848Arg variant.^38^ Overall, 48% (13/27) of the patients with missense variants in the 844–848 region in the present cohort had a p.Gly848 missense variant, compared with only 22% (36/162) in the study by Koczkowska *et al*^12^ describing a majority of variants affecting p.Leu847 (48%, 78/162). Functional studies suggested that mutations within this region may destabilise neurofibromin via protein misfolding, leading to dominant-negative effects.^35 39^

Variants at p.Arg1276 and p.Lys1423, both in the GRD, were clearly linked to more severe NF1, including skeletal anomalies, Noonan-like features and pulmonic stenosis.^11^ In our cohort, NF1 patients with p.Arg1276 missense variants showed significantly fewer cNFs, associated with a more severe form of NF1 with more frequent spinal NFs, bone abnormalities, Noonan-like features and cardiovascular abnormalities (table 3 and figure 2); pNFs were also significantly more frequent, in contrast to the observations of Koczkowska *et al* (6/11, 55% vs 5/64, 8%, p=0.014 after correction for multiple testing).^11^ Finally, p.Lys1423 missense variants were associated with more severe NF1, with more frequent plexiform and spinal NFs, bone abnormalities, Noonan-like features, cardiovascular abnormalities and cognitive impairment or learning difficulties (table 4 and figure 2). In addition, we describe a lower frequency of Lisch nodules and cNFs, which has not been reported to date in this group of patients. Arg1276 and Lys1423 are localised in the GRD.^40^ p.Arg1276 and p.Lys1423 missense variants were associated with a significant increase in the activated intracellular form of RAS-GTP.^35 37 41^

p.Met1149 variants were infrequent in our series, but the available data point to a mild phenotype with few neurofibromas (online supplemental table 2 and figure 2). This variant lies in the tubulin-binding domain, possibly involved in neurofibromin dimerisation.^42^ Conversely, p.Arg1204 missense variants, though rare, were consistently associated with absence of neurofibromas, and more frequent short stature, learning difficulties and Noonan-like features. We report here a milder phenotype for patients with missense variants at p.Arg1204 of neurofibromin, though with a limited number of cases (0.20%, 7/3479 of all positive index cases). Especially, none of the patients developed MPNST, the major and most feared complication in NF1. These observations remain to be replicated in independent studies but suggest a possible hypomorphic variant.

Other rare variants previously reported include p.Arg1038Gly, associated with a mild phenotype.^19^ Given the limited number of patients described with this variant, only a larger cohort analysis could confirm these initial observations: no patient in our cohort exhibited the p.Arg1038Gly variant. A more recent correlation reported the association between splice variants leading to skipping of exon 24 and a mild phenotype with absence of neurofibromas.^25^ We estimate the prevalence of such variants in positive index cases to be about 0.20% (7/3479) in the French cohort (no comprehensive phenotypic data for these variants).

The genotype–phenotype correlations of several *NF1* variants with a specific form of NF1 have been facilitated by the relative recurrence of these variants and by international collaborative studies. Other correlations certainly exist, but for more uncommon variants that simply cannot be assessed individually in relatively small cohorts of patients. To identify these variants, two approaches are possible: (1) set up a large European or international cohort (with the involvement of patient associations), which would then require exhaustive, standardised phenotyping which could be a limiting factor depending on the expertise of the contributing centres; (2) evaluate variants statistically in groups, which has not been conclusive in separating missense variants from truncating variants, but which could prove relevant by focusing on missense variants localised in well-defined functional domains of neurofibromin. In vitro and in vivo models, including organoids, will be critical for delineating whether a given variant acts through a hypomorphic or dominant-negative mechanism. Such studies will refine prognostic predictions and patient management strategies in NF1.

Our findings reinforce the clinical relevance of several NF1 point variants and support a shift towards genotype-guided care in NF1.

## Methods

### Study cohort

1635 NF1 patients were enrolled between 2002 and 2013 in three French clinical research programmes as part of the NF-France database.^6^ In addition, databases from routine molecular diagnosis were retrospectively analysed. Between 2013 and 2020, 4091 index cases with a clinical presentation suggestive of NF1 were molecularly screened for NF1 in the Cochin Hospital (Assistance Publique-Hôpitaux de Paris, Paris, France). Nine patients were also included from the Radboud University Medical Centre (the Netherlands), and three from the Maastricht University Medical Center (the Netherlands).

### Phenotypes

Phenotypical data were recorded with a standardised questionnaire. Patients with missing data were considered as ‘not specified’ for the trait and were not included in the statistical analysis for that trait. Most features were identified by physical examination, with Lisch nodules being diagnosed, or excluded, by slit-lamp examination; individuals not given a slit lamp examination were coded as ‘not documented’ (ND) and excluded from further analysis of the trait. The presence or absence of OPGs was determined by cranial MRI or CT examination, with individuals not given cranial imaging being encoded as ‘ND’. Facial dysmorphism was evaluated on the following aspects: coarse face, flat occiput/brachycephaly, facial asymmetry, prominent forehead, frontal bossing, ptosis, hypertelorism, midface hypoplasia, triangular face, small and down-slanting palpebral fissures, eversion of the lateral eyelid, epicanthic folds, telecanthus, large and low set ears, high and broad nasal bridge, wide and prominent philtrum, micrognathia, small pointed chin, low posterior hairline, webbed neck. Stature and weight were evaluated according to the 2018 AFPA (French association of ambulatory pediatrics) reference curves. Head circumference was evaluated according to the 2018 AFPA reference curves for children under 5 years old and according to the Nellhaus charts when older. Short and tall stature were defined as a height that was more than two SD, respectively, below or above the age-matched and sex-matched population mean. Macrocephaly was defined as a head circumference that was more than 2 SD above the age-matched and sex-matched population mean. The diagnosis of learning disabilities was performed on specific testing of cognitive abilities and/or a history of scholar difficulties leading to repeating at least one level. As the small number of observations of each individual malignancy would have resulted in a lack of power in statistical analyses, we pooled all types of malignant tumours into a single item (namely ‘malignancies’) and performed analyses on this composed trait.

### Molecular analysis

The molecular analysis of the *NF1* gene was performed using a variety of screening methodologies including DNA and RNA sequencing, polymorphic microsatellite marker analysis and multiplex ligation-dependent probe amplification, comparative genomic hybridisation array or real-time PCR-based gene-dosage analysis to permit deletion assessment, as previously described.^43^,^45^ Assessment of variant implications was performed based on population databases (dbSNP and gnomAD), variant databases (HGMD, LOVD and COSMIC) and prediction software (Alamut and mutation taster). An assessment of variants’ pathogenicity was performed according to the American College of Medical Genetics and Genomics and the Association for Molecular Pathology guidelines.

### Statistical analyses

Univariate analysis was performed using a two-tailed Fisher’s exact test to compare categorical variables. Resulting p values were adjusted using the Benjamini-Hochberg correction for multiple comparisons.^46^ Reference cohort for comparison (‘classic NF1’) was obtained from Koczkowska *et al*^11^ Statistical analyses were performed with the R stats package in RStudio V.4.0.3. Radar charts were obtained with the fmsb package in RStudio V.4.0.3.

## Supplementary material

10.1136/jmg-2025-110783 online supplemental file 1

## Data Availability

All data relevant to the study are included in the article or uploaded as online supplemental information.
