# Supplementary material for: Refined genotype–phenotype correlations in neurofibromatosis type 1 patients with NF1 point variants
Source: J Med Genet. 2025 Aug 4;62(12):e110783. doi: 10.1136/jmg-2025-110783 (PMC12703300; doi:10.1136/jmg-2025-110783)
Supplement: online supplemental file 1 [file jmg-62-12-s001.pdf]

Supplemental Table 1. Clinical findings in patients with *NF1* point variants associated with specific forms of NF1.

	**p.Met992del**		**p.Arg1809**		**Codons 844-848**		**p.Met1149**		**p.Arg1276**		**p.Lys1423**		**Skipping exon 24**	**p.Arg1204**	Frequency in NF1 population from Koczkowska *et al.* 2020[11]
	Upadhyaya *et al.* 2007[9]	Koczkowska *et al.* 2019[10]	Rojnueangnit *et al.* 2015[13]	Present study	Koczkowska *et al.* 2018[12]	Present study	Koczkowska *et al.* 2020[11]	Present study	Koczkowska *et al.* 2020[11]	Present study	Koczkowska *et al.* 2020[11]	Present study	Chen *et al.* 2024[31]	Present study	
Number of patients [families]	47 [21]	135 [103]	136 [98]	24 [13]	162 [129]	27 [17]	69 [50]	2 [2]	119 [101]	25 [22]	93 [86]	35 [29]	40 [26]	11 [8]	
CALS	47/47 (100%)	165/182 (90.7%)	124/136 (91.2%)	20/24 (83%)	130/157 (82.8%) ↘	22/27 (81%)	62/69 (89.9%)	2/2 (100%)	111/119 (93.3%)	23/25 (92%)	86/91 (94.5%)	30/35 (86%)	34/40 (85.0%)	10/11 (91%)	1537/1728 (89%)
Freckling	30/47 (63.8%)	105/171 (61.4%) ↘	54/80 (67.5%)	14/23 (61%) ↘*	104/144 (72.2%) ↘	19/26 (73%)	40/65 (61.5%) ↘	2/2 (100%)	74/112 (66.1%) ↘	19/24 (79%)	65/85 (76.5%)	24/34 (71%)	23/38 (60.5%) ↘	3/6 (50%)	1403/1667 (84.2%)
Lisch nodules	3/38 (7.9%)	16/139 (11.5%) ↘	10/119 (8.4%) ↘	3/19 (16%) ↘*	42/98 (42.9%) ↘	10/15 (67%)	3/44 (6.8%) ↘	0/1 (0%)	19/70 (24.1%) ↘	3/11 (27%)	31/59 (52.5%)	8/27 (30%) ↘*	0/23 (0%) ↘	0/4 (0%) ↘°	729/1237 (58.9%)
cNFs[a]	0/18 (0%) ↘	0–1/57 (0–1.8%) ↘	5/59 (8.5%) ↘	1/10 (10%) ↘*	47/69 (68.1%) ↘	12/14 (86%)	0–3/24 (0–12.5%) ↘	0/2 (0%)	14/40 (35%) ↘	6/13 (46%) ↘*	23/28 (82.1%)	12/16 (75%)	0/8 (0%) ↘	0/1 (0%)	656/723 (90.7%)
scNFs[a]		0–3/36 (0–8.3%) ↘		0/10 (0%) ↘*	33/65 (50.8%)	9/14 (64%)	0–3/22 (0–13.6%) ↘	0/2 (0%)	21/37 (56.8%)	9/13 (69%)	13/23 (56.5%)	11/15 (73%)	0/8 (0%) ↘	0/1 (0%)	297/515 (57.7%)
pNFs[b]	0/41[#] (0%) ↘	0/125[#] (0%) ↘	0/107[#] (0%) ↘	0/2 (0%)	36/92[#] (39.1%) ↗	6/15 (40%) ↗°	0/42[#] (0%) ↘		5/64[#] (7.8%)	6/11 (55%) ↗*	14/48[#] (29.2%)	12/19 (63%) ↗*	0/18[#] (0%)	0/1 (0%)	120/648[#] (18.5%)
Symptomatic spinal NFs (all ages)	1/47 (2.1%)	1/165 (0.6%)		0/2[b] (0%)	13/127[b] (10.2%) ↗	1/11[b] (9%)	0/59 (0%)		18/97 (18.6%) ↗	3/6[b] (50%) ↗*	3/65 (4.6%)	2/11[b] (18%) ↗*	0/33 (0%)	ND	36/2058 (1.8%)
Symptomatic OPGs		0/170 (0%) ↘	0/119 (0%) ↘	0/13 (0%)	12/136 (8.8%) ↗	0/17 (0%)	0/58 (0%)		0/97 (0%)	0/16 (0%)	1/74 (1.4%)	2/23 (9%)	0/37 (0%)	0/5 (0%)	64/1650 (3.9%)
Asymptomatic OPGs		1/41 (2.4%) ↘		0/13 (0%)	18/63 (28.6%) ↗	1/17 (6%)	0/23 (0%)		1/48 (2.1%)	0/16 (0%)	6/40 (15%)	2/23 (9%)		0/5 (0%)	70/519 (13.5%)
Other cancers		6/126 (4.8%) (brain tumors)		0/22 (0%)	13/139 (9.4%) ↗	2/25 (8%)	0/57 (0%)	0/2 (0%)	4/94 (4.3%)	0/22 (0%)	7/77 (9.1%)	1/28 (4%)		0/7 (0%)	18/523 (3.4%)
Musculokeletal abnormalities		30/172 (17.4)		8/15 (53%) ↗*	48/144 (33.3%) ↗	12/19 (63%) ↗*	15/61 (24.6%)	0/2 (0%)	32/100 (32%) ↗	8/18 (44%) ↗*	34/83 (41%) ↗	16/23 (70%) ↗*	4/36 (11.1%)	0/1 (0%)	144/948 (15.2%)
Scoliosis[a]	2/20 (10%)	7/57 (12.3%)	6/71 (8.5%) ↘	3/9 (33%)	20/64 (31.3%)	8/14 (57%) ↗°	2/20 (10%)	0/2 (0%)	8/35 (22.9%)	3/14 (21%)	10/27 (37%)	11/14 (79%) ↗*	2/36 (5.6%) ↘	0/1 (0%)	51/236 (21.6%)
Pectus abnormalities	7/45 (15.6%) ↘		9/125 (7.2%) ↘												
Noonan-like features		19/166 (11.5%) ↗	32/122 (26.2%)	9/23 (39%) ↗*	10/134 (7.5%) ↗	3/24 (13%) ↗°	18/62 (29%) ↗	0/2 (0%)	22/106 (20.8%) ↗	7/24 (29%) ↗*	24/83 (28.9%) ↗	7/27 (26%) ↗*	1/35 (2.9%)	2/4 (50%) ↗*	57/1683 (3.4%)
Pulmonary stenosis	4/47 (8.5%) ↗	8/160 (5%) ↗	13/105 (12.4%) ↗	1/19 (5%)	2/113 (1.8%)	0/9 (0%)	2/52 (3.9%)	0/1 (0%)	11/92 (12%) ↗	4/15 (27%) ↗*	11/76 (14.5%) ↗	1/15 (7%)			25/2322 (1.1%)
Short stature	5/47 (10.6%) ↘	16/118 (13.6%)	29/82 (35.4%) ↗	2/20 (10%)	15/91 (16.5%)	6/24 (25%)	5/33 (15.2%)	0/2 (0%)	14/80 (17.5%)	4/22 (18%)	21/51 (41.2%) ↗	7/31 (23%)		4/6 (67%) ↗*	109/684 (15.9%)
Macrocephaly	4/45 (8.9%) ↘	30/132 (22.7%) ↘	20/80 (25%)	4/18 (22%)	36/98 (36.7%)	6/20 (30%)	19/45 (42.2%)	1/1 (100%)	24/76 (31.6%)	5/17 (29%)	15/51 (29.4%)	9/27 (33%)		2/4 (50%)	239/704 (33.9%)
Developmental disorders and/or learning difficulties	8/47 (17.0%) ↘	58/176 (33%) ↘	71/127 (55.9%)	14/22 (64%)	56/138 (40.6%)	14/26 (54%)	31/66 (47%)	0/2 (0%)	46/105 (43.8%)	11/25 (44%)	36/87 (41.4%)	21/33 (64%) ↗°	13/39 (33.3%)	6/6 (100%) ↗*	190/424 (44.8%)
Cardiovascular abnormalities				2/19 (11%)		0/22 (0%)	5/52 (9.6%)	0/1 (0%)	22/92 (23.9%) ↗	5/23 (22%) ↗*	19/76 (25%) ↗	6/27 (22%) ↗*		1/3 (33%)	54/2.322 (2.3%)

[a]patients ≥19 years old; [b]patients ≥9 years old; [#]externally visible pNFs. The frequency of clinical traits with a circle (°) or asterisk (*) significantly differs from the "classic" NF1 cohort, respectively only before or after correction for multiple testing using the Benjamini-Hochberg (B-H) procedure. The direction of the arrow indicates when the clinical trait is more frequent (↗) or less frequent (↘) compared to the "classic" NF1 cohort. NFs: neurofibromas; cNFs: cutaneous neurofibromas; scNFs: subcutaneous neurofibromas; pNFs: plexiform neurofibromas; CALS: café-au-lait spots; OPGs: optic pathways gliomas; ND: not documented.

Supplemental Table 2. Clinical data in two NF1 patients with Met1149 missense variants.

	Age: 9-18	Age >18	Total n/N*	Total %
Age range (years)	11	22	11-22	
Median age (years)	11	22	16.5	
Number of individuals (index cases:relatives)	1:0	1:0	2:0	
Male:Female	1:0	1:0	2:0	
Clinical diagnostic criteria fulfilled considering family history	1/1	1/1	2/2	100%
Clinical diagnostic criteria fulfilled regardless family history	1/1	1/1	2/2	100%
Café-au-lait spots[a]	1/1	1/1	2/2	100%
1-5				
6-100	1/1	1/1	2/2	
>100				
Freckling	1/1	1/1	2/2	100%
Blue-red macules	0/1	ND	0/1	0%
Lisch nodules	ND	0/1	0/1	0%
Cutaneous neurofibroma[b]	0/1	0/1	0/2	0%
Subcutaneous neurofibroma[b]	0/1	0/1	0/2	0%
Deep neurofibroma	ND	ND	ND	
Plexiform neurofibroma	ND	ND	ND	
Spinal neurofibroma	ND	ND	ND	
Optic pathway glioma[c]	ND	ND	ND	
Other tumors	0/1	0/1	0/2	0%
Musculoskeletal abnormalities	0/1	0/1	0/2	0%
Noonan-like features	0/1	0/1	0/2	0%
Short stature (<2 sd)	0/1	0/1	0/2	0%
Macrocephaly (>2 sd)	ND	1/1	1/1	100%
Neurological abnormalities	0/1	0/1	0/2	0%
Cognitive disorders and/or learning difficulties	0/1	0/1	0/2	0%
Cardiovascular abnormalities	0/1	ND	0/1	0%

*n=number of patients fulfilling the criterion; N=total number of patients for whom data were available. [a]All sizes; [b]Including non-histologically confirmed neurofibromas; [c]Optic pathways gliomas identified by brain MRI or CT-scan. MPNSTs: malignant peripheral nerve sheath tumors; UBOs: unidentified bright objects (hyperintense regions seen on T2-weighted magnetic resonance brain scans); ND: not documented.

Supplemental Table 3. Clinical data in NF1 patients with Arg1204 missense variants.

	**Age: 0-8**	**Age: 9-18**	**Age >18**	**Total n/N***	**Total %**
Age range (years)	4 months-8 years		29-57	4 months-57 years	
Median age (years)	4		35	6	
Number of individuals (index cases:relatives)	7:0		1:3	8:3	
Male:Female	4:3		2:2	6:5	
Clinical diagnostic criteria fulfilled considering family history	6/7		3/4	9/11	82%
Clinical diagnostic criteria fulfilled without considering family history	3/7		2/4	5/11	45%
Café-au-lait spots[a]	7/7		4/4	11/11	100%
1-5			1/4		
6-100	5/7		1/4		
>100					
Not quantified	2/7		2/4		
Freckling	2/5		1/1	3/6	50%
Blue-red macules	0/4		ND	0/4	0%
Lisch nodules	0/3		0/1	0/4	0%
Cutaneous neurofibroma[b]	0/5		0/1	0/6	0%
Subcutaneous neurofibroma[b]	0/5		0/1	0/6	0%
Plexiform neurofibroma	0/4		0/1	0/5	0%
Spinal neurofibroma	0/2		ND	0/2	0%
Optic pathway glioma[c]	0/5		ND	0/5	0%
Other tumors	0/5		0/2	0/7	0%
Musculoskeletal abnormalities[d]	2/4		0/1	2/5	20%
Scoliosis	0/4				
Pectus abnormalities	0/4				
Noonan-like features	2/4		ND	2/4	50%
Short stature (<2 SD)	3/5		1/1	4/6	67%
Macrocephaly (>2 SD)	2/4		ND	2/4	50%
Neurological abnormalities[e]	4/5		ND	4/5	80%
UBOs	0/5				
Cognitive disorders and/or learning difficulties	4/5		2/2	6/7	86%
Cardiovascular abnormalities[f]	1/3		ND	1/3	33%

*n=number of patients fulfilling the criterion; N=total number of patients for whom data were available. [a]All sizes; [b]Including non-histologically confirmed neurofibromas; [c]OPGs identified by brain MRI or CT-scan; [d]Musculoskeletal abnormalities including large hands and feet, joint hyperlaxity, L2-L3 hemivertebrae; [e]Neurological abnormalities including ventricular enlargement, epilepsy, optic pathway atrophy, hypotonia; [f]Cardiovascular abnormalities including pulmonary valve stenosis. MPNSTs: malignant peripheral nerve sheath tumors; UBOs: unidentified bright objects (hyperintense regions seen on T2-weighted magnetic resonance brain scans); ND: not documented.
